# The chemical nature of phenolic compounds determines their toxicity and induces distinct physiological responses in *Saccharomyces cerevisiae* in lignocellulose hydrolysates

**DOI:** 10.1186/s13568-014-0046-7

**Published:** 2014-05-29

**Authors:** Peter Temitope Adeboye, Maurizio Bettiga, Lisbeth Olsson

**Affiliations:** 1 Industrial Biotechnology, Department of Chemical and Biological Engineering, Chalmers University of Technology, Gothenburg SE-412 96, Sweden

**Keywords:** Phenolics, Toxicity, Inhibition, Tolerance, Conversion, Saccharomyces cerevisiae

## Abstract

We investigated the severity of the inhibitory effects of 13 phenolic compounds usually found in spruce hydrolysates (4-hydroxy-3-methoxycinnamaldehyde, homovanilyl alcohol, vanillin, syringic acid, vanillic acid, gallic acid, dihydroferulic acid, *p*-coumaric acid, hydroquinone, ferulic acid, homovanillic acid, 4-hydroxybenzoic acid and vanillylidenacetone). The effects of the selected compounds on cell growth, biomass yield and ethanol yield were studied and the toxic concentration threshold was defined for each compound. Using Ethanol Red, the popular industrial strain of *Saccharomyces cerevisiae*, we found the most toxic compound to be 4-hydroxy-3-methoxycinnamaldehyde which inhibited growth at a concentration of 1.8 mM. We also observed that toxicity did not generally follow a trend based on the aldehyde, acid, ketone or alcohol classification of phenolic compounds, but rather that other structural properties such as additional functional groups attached to the compound may determine its toxicity. Three distinctive growth patterns that effectively clustered all the compounds involved in the screening into three categories. We suggest that the compounds have different cellular targets, and that. We suggest that the compounds have different cellular targets and inhibitory mechanisms in the cells, also compounds who share similar pattern on cell growth may have similar inhibitory effect and mechanisms of inhibition.

## Introduction

Lignocellulose, primarily made up of carbohydrates and lignin, has been billed as the most abundant material on earth (Chandel *et al*. [2011]). Next to carbohydrates, aromatic compounds are the second most abundant class of organic compounds in nature (Boll *et al*. [2002]). It has been claimed that aromatic compounds, including phenolics make up about 25% of the earth’s biomass (Gibson and Harwood [2002]). This abundance is significant to the usage of plants and plant residues as important resources in second generation biofuel and chemicals production.

Phenolic compounds are secondary metabolites that are synthesized by plants via the pentose phosphate, shikimate and phenylpropanoid pathways (Randhir *et al*. [2004]). They form the building blocks of lignin and they play crucial role in plants resistance to diseases and infections (Hutzler *et al*. [1998], Nicholson and Hammerschmidt [1992], Vance *et al*. [1980], Vanholme *et al*. [2010]). Lignin in itself is a natural polymer that is primarily made up of phenylpropane units derived from guaiacol, p-hydroxyphenol and syringol, all interconnected in a C-C bond (Dorrestijn *et al*. [2000], Mcdonough [1983], Nenkova et al. [2011]). Phenolic compounds are directly involved in various plant physiological processes and plant defense mechanisms against microbial infections (Bhattacharya *et al*. [2010], Blum *et al*. [1999], Bravo [1998], Hutzler *et al*. [1998], Muller *et al*. [1964]). In addition, their antimicrobial, antioxidant activity, and their various other dietary and pharmaceutical properties make them highly relevant to food and pharmaceutical industries (Balasundram *et al*. [2006], Benavente-Garcia *et al*. [1997], Hertog *et al*. [1993], Puupponen-Pimia *et al*. [2001], Scalbert and Mazur [2002]). On the other hand, the beneficial effect of the antimicrobial activities of phenolic compounds which is beneficial to plants present a significant challenge to the production of second generation bioethanol and other chemicals from plant residues and lignocellulosic materials (Klinke *et al*. [2004]). During biofuel production, plant biomasses are first subjected to pre-treatment processes and hydrolysis in order to breakdown their structures and adapt them to forms accessible by enzymes for fermentation and bioconversion. Diverse phenolic compounds are formed as residues of lignin degradation during these wood and plant residue pre-treatment processes for hydrolysate production and wood pulping (Guss [1945], Klinke *et al*. [2004], Larsson *et al*. [2000], Larsson *et al*. [1999b], Taherzadeh and Karimi [2007]). The composition of the different phenolic compounds formed during pre-treatment varies and depend on both the plant source and the pre-treatment method (Larsson *et al*. [1999b]). In general, the resulting mix is usually made up of phenolic acids, phenolic aldehydes, phenolic alcohols and phenolic ketones all of which are inhibitory to cells. A typical spruce hydrolysate will often consist of the phenolic compounds listed in Table 1.

**Table 1:** Table of phenolic compounds and the concentration range commonly found in spruce hydrolysates

**Phenolic compounds**	**Amount (mg/L)**
Gallic acid	7.1–10.2
Catechine	61–71.9
Vanillic acid	3.93–71.2
Syringic acid	42.3–42.87
Ferulic acid	42.91–45.08
Picein [3-(β-d-glucosyloxy)-hydroxy-acetophenone]	0.2–1.4
Pungenin[3-(β-d-glucosyloxy)-4-hydroxy-acetophenone]	0.2
Taxifoloin	2–33
Coniferyl aldehyde	35–301
Vanillic acid	0.01–35
Vanillin	36
4-hydroxybenzoic acid	39–81
Catechol	2
Acetoguaiacone	146
Trans cinnamic acid	10
Syringaldehyde	107

(Almeida et al. [2007b], Deflorio *et al*. [2011], Delvas *et al*. [2011], Evensen *et al*. [2000], Hutzler *et al*. [1998], Miyafuji *et al*. [2003]).

Also, Pungenol (3′,4′-hydroxy-acetophenone), Piceol (4′-hydroxyacetophenone), Trans-resveratrol, _P_-Coumaric acid, Coumarins, Stilbenes, Styryl pyrones, Dihydroconiferyl alcohol, Hydroquinone, Homovanillic acid have all been found in various concentrations in spruce hydrolysates (Almeida et al. [2007b], Deflorio *et al*. [2011], Delvas *et al*. [2011], Evensen *et al*. [2000], Hutzler *et al*. [1998], Miyafuji *et al*. [2003]).

The occurrence of phenolic compounds with various functional groups like aldehydes, acids, ketone and alcoholic, and the abundance of phenolic compounds in wood hydrolysates present major challenges to studying them in detail. In some studies aimed at understanding phenolic compounds, compounds having similar functional groups have been grouped together while representative compounds of each group were studied (Larsson *et al*. [2000]), presumably under the assumption that compounds having the same functional group are similar in their inhibitory activities. It has been shown that the presence of phenolic compounds in hydrolysates may determine the fermentability of hydrolysates and directly impacts on ethanol productivity of *S. cerevisiae* (Larsson *et al*. [1999a], Larsson *et al*. [2000]). The effects of many selected phenolic compounds and other inhibitors on yeast fermentative conditions have been screened, and strains of *S. cerevisiae* engineered for phenolic tolerance have been constructed and evaluated (Delgenes *et al*. [1996], Gregg and Saddler [1996], Larsson *et al*. [2000]). It is known that certain phenolic compounds such as ferulic acid and vanillin can be assimilated and converted by *S. cerevisiae* (Clausen *et al*. [1994], Huang *et al*. [1993], Vanbeneden *et al*. [2008]) however there are concentrations at which *S. cerevisiae* cannot survive the inhibition of such compounds, the various concentrations have not been defined for phenolic compounds.

Basing our experimental work on the hypotheses that (i) different phenolic compounds have different limits of toxicity on *S. cerevisiae* and (ii) mechanisms and activities of inhibition among phenolic compounds may be compound-specific, we have defined the toxicity limits of 13 different phenolic compounds selected from all classes of phenolic compounds commonly found in but not limited to spruce hydrolysates. We also studied the effects of the various phenolic compounds on the growth of *S. cerevisiae* and categorised the phenolic compounds into clusters based on their effects on growth. The influence of each cluster of phenolic compounds on metabolite yields were investigated in order to draw parallels and similarities between the phenolic compounds within each cluster and to explore whether compounds within each cluster have similar influence on the physiology of *S. cerevisiae*, all in order to better understand phenolic inhibition in lignocellulosic fermentation.

## Materials and methods

### Yeast strain

The industrial yeast strain *S. cerevisiae* Ethanol Red was used for this study. The yeast strain was obtained from the local wine-making and brewery shop in Gothenburg, Sweden.

### Reagents

4-hydroxy-3-methoxycinnamaldehyde, homovanilyl alcohol, vanillin, syringic acid, vanillic acid, gallic acid, dihydroferulic acid, *p*-coumaric acid, hydroquinone, ferulic acid, homovanillic acid, 4-hydroxybenzoic acid and vanillylidenacetone and other reagents used for growth media preparation in the studies were procured from Sigma-Aldrich.

### Preparation of culture media

The medium used for all the cultivations was Yeast Minimal Mineral Medium (YMMM) (Verduyn *et al*. [1992]). YMMM containing single phenolic substrates was prepared for each phenolic compound using the concentrations reported under “Results”.

### High throughput toxicity screening of phenolic compounds on *S. cerevisiae*

To define the range of values within which the toxicity limits of the compounds lie, high throughput toxicity screenings were done using Bioscreen C MBR (Oy Growth Curves Ab Ltd, Finland). Several concentrations of each phenolic compound were tested. Five replicates of each concentration step were run in parallel in the following conditions: T = 30°C ± 0.1; time = 96 hours; shaking speed setting = “maximum” optical density (OD) reading period = 15 min; wavelength filter = wideband 450 – 580 nm; initial OD = 0.1.

To facilitate data comparison, the readings obtained from the bioscreen were calculated back to standard spectrophotometric measurements at 600 nm via the formula:

(1) $$OD_{\mathrm{spectro}}=\frac{OD_{\mathrm{Bioscreen}}}{\mathrm{Path}\;\mathrm{Length}\left(\mathrm{cm}\right)\times 1.32}$$

Where

 OD_spectro_ = equivalent OD on spectrophotometer at 600 nm

 OD_Bioscreen_ = measured OD on the bioscreen

(2) $$\mathrm{PathLength}=\frac{\mathrm{volume}\;\left(\mathrm{ml}\right)}{r^{2}\;X\;\pi }$$

Where: volume = culture volume in a well in the bioscreen plate; r = radius of the well.

Non-linearity at higher cell densities was corrected as described by Warringer *et al.*, (Warringer and Blomberg [2003]) using the formula:

(3) $$\mathrm{ODcor}=\mathrm{ODobs}+\left(OD^{2}\mathrm{obs}*0.449\right)+\left(OD^{3}\mathrm{obs}*0.191\right)$$

Where: ODcor = the corrected OD and ODobs = the observed OD values, from which the average blank has been subtracted.

Aerobic batch cultivations were carried out in 100 ml or 250 ml baffled Erlenmeyer flasks (SIMAX, Czech Republic), containing 20 ml and 50 ml medium, respectively.

### OD measurement of culture

Growth measurement for shake flask cultivations was done by measuring the turbidity of the culture at A_600nm_ using a Thermo Scientific GENESYS 20 Visible Spectrophotometer.

### Determination of dry cell weight

Determination of Dry Cell Weight (DCW) was done in duplicates. Cells were harvested by filtration using pre-dried and weighed filter paper discs of 0.45 μm pore size (Sartorius Stedim Biotech, Goettingen, Germany) on a water tap vacuum filter unit (Sartorius Stedim Biotech, Goettingen, Germany). The filter paper discs were dried in a microwave at 120 W for 15 minutes, weighed again and the biomass concentrated was calculated from the difference. DCW data were used for the calculation of biomass yield.

### Analysis of metabolites

Analysis of ethanol, glycerol and acetate from the cultivation was performed by high performance liquid chromatography (HPLC) using a Dionex Ultimate 3000 HPLC unit (Thermo scientific, Dionex Corporation, Sunnyvale, USA) equipped with an Aminex HPX-87H (Biorad, USA) column of length 300 mm and diameter 7.8 mm, which was packed with 9 μm particles. A column temperature of 45°C was used for analysis and 5 mM H2SO4 was used as the mobile phase with a flow rate of 0.6 ml/min throughout the analysis. A Shodex RI-101 RI detector and an Ultimate 3000 VWD 3100 variable wavelength ultraviolet detector coupled to the HPLC unit were used to quantify the metabolites.

### Determination yields

Yields (Yi/s) of ethanol, glycerol, acetate and biomass from the total consumed substrate (glucose) were calculated during the exponential growth phase by plotting each of the products against the total consumed glucose. The yield for each product was then obtained as the slope of the plot. Average values of biological replicates were used as the final yield for each culture condition.

### Establishing concentration ladder of compounds

A concentration series was set up in increasing order for each compound to be screened for effect on *S. cerevisiae*. Since toxicity varies widely and using a universal concentration series for all of the compounds was not feasible, we determined consistent ratios of increase between consecutive points in the concentration series for all compounds to allow comparison of toxicity among the various compounds.

### Determination of toxicity limits

The toxicity limits of the different phenolic compounds were determined based on the aspect (maximum specific growth rates or final OD or elongation of lag phase) at which the yeast cultivations were most affected. The maximum specific growth rate of the *S. cerevisiae* in the presence of increasing concentrations of phenolic compounds were determined with increasing concentration of the compounds until cell growth stopped.

### Statistical validation of data

All experimental data were subjected to Student t-test to determine the significance level with respect to the control. The number of replicates varied from 3 to 7, depending on the experiment. Therefore, t-tests for two-samples assuming unequal variances were performed with a significance level of probability set at p < 0.05. All error bars were standard deviations of multiple measurements of each parameter, all derived from biological replicates.

## Results

### Effect of compounds on *S. cerevisiae* growth pattern

**Figure 1:**
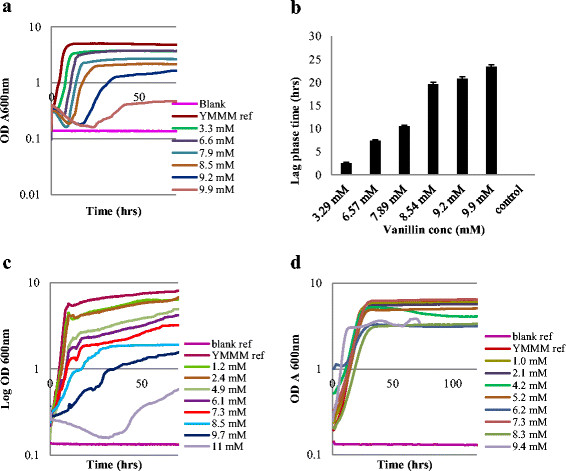
**Three distinct growth profiles of*Saccharomyces cerevisiae*in the presence of phenolic compounds with: a. vanillin; c.*p*-Coumaric acid; d. Vanillylidenacetone representing common growth profiles within the three cluster groups.** Elongation in lag phase was only shown in figure **b** for the first cluster since there was no lag phase elongation in the second and third clusters of compounds.

We hypothesized that the physiological effect of each phenolic compound on *S. cerevisiae* would be unique and have phenotypic traits demonstrated in the growth pattern of *S. cerevisiae*. Yeast was grown in the presence of 4-hydroxy-3-methoxycinnamaldehyde, homovanilyl alcohol, p-coumaric acid, hydroquinone, ferulic acid, vanillin, syringic acid, homovanillic acid, 4-hydroxybenzoic acid, vanillic acid, gallic acid, vanillylidenacetone and dihydroferulic acid and the impact of the compounds on growth profiles, maximum specific growth rates and culture turbidity was assessed. *S. cerevisiae* had three unique growth patterns that distinctively grouped all the phenolic compounds into three clusters which we named cluster 1, cluster 2 and cluster 3 (Figure 1).

The culture containing phenolic compounds in cluster 1, exhibited lag phase elongation which increased with increasing concentration of phenolic compounds in the medium until a concentration of compound is attained at which growth was no longer possible. The second and third growth pattern clusters showed no elongation of lag phase. The phenolic compounds also had specific effects on maximum specific growth rate within their clusters. In cultures containing cluster 1 compounds, maximum specific growth rate decreased with increasing concentration of phenolic compound until a concentration is attained at which growth finally stopped (Additional file 1: Figure S1). Similarly, in cultures containing the cluster 2 compounds, the maximum specific growth rate reduced with increasing concentration of phenolic compound until a concentration is attained at which growth was no longer possible (Additional file 1: Figure S1b). In cultures contaning the third cluster of compounds however (as illustrated in Additional file 1: Figure S1c), the maximum specific growth rate remained constant until the concentration was attained at which growth was no longer possible.

The determination of biomass formation in the cultivations was limited to OD measurements on the bioscreen. In cultivations containing cluster 1 compounds, the OD of the yeast cultivations decreased with increasing concentration of phenolic compound until a concentration is attained at which growth stopped. As illustrated in Additional file 2: Figures S2a and S2, the reduction in OD was observed to be valid for the first and second clusters of compounds. In the third cluster however, although a reduction in the final OD was observed (Additional file 2: Figure S2c), the observed reduction was not as strong as in the first two compound clusters.

This categorization growth profile groups the thirteen phenolic compounds as;

 Cluster1: 4-hydroxy-3-methoxycinnamaldehyde, Homovanilyl alcohol, Vanillin, Syringic acid and Dihydroferulic acid.

 Cluster 2: p-Coumaric acid, hydroquinone, Ferulic acid, Homovanillic acid and 4-hydroxybenzoic acid and

 Cluster 3: Vanillic acid, Gallic acid and Vanillylidenacetone.

### Different phenolic compounds have different limits of toxicity

**Figure 2:**
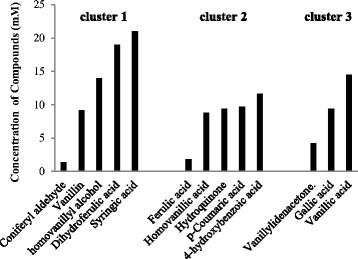
**Bar chart showing concentrations at which different phenolic compounds within each growth-based cluster became too toxic for the growth of** ***Saccharomyces cerevisiae*.**

In experimentally defining the concentration threshold at which the selected phenolic compounds completely inhibit yeast growth, we conducted toxicity screening on the phenolic compounds. During the screening, the maximum specific growth rates of the cultures when growth was last observed ranged between 0.07 h^−1^ and 0.09 h^−1^, this was about 20% of the maximum specific growth rate of the control. In the presence of another set of phenolic compounds in which the cells experienced increased lag phase and reduced biomass with increasing concentration of the phenolic compounds, the concentration at which the cells last showed observable growth had an elongated the lag phase of about 5 times that of the control, the cells stopped growing in higher concentrations. In the third category, the cells suddenly stopped growing after a certain concentration and this concentration was noted. The toxicity screening revealed a wide range of toxicity among the compounds (Figure 2). The screening also revealed that each compound has a toxicity limit that is not necessarily based on its classification as an acid, alcohol, aldehyde or a ketone. Coniferyl aldehyde had the highest toxicity, becoming extremely toxic at 1.4 mM for cells to grow while syringic acid is the least toxic with cell growth continuing to be recorded at 22 mM. Further concentration increase in syringic acid was limited by strong interference in measurements as a result of the deep colouration of the medium (Figure 2).

### Effects of toxic concentrations of phenolic compounds on ethanol and biomass titres and yields

In the next step we investigated whether compounds clustered together by order of growth pattern would also have similar effect on the yeast cell physiology. A pair of compounds was selected from each cluster and their effects on ethanol, acetate, glycerol and biomass yields were determined. Syringic and dihydroferulic acids were selected from the first cluster, homovanillic and 4-hydroxybenzoic acids were selected from the second cluster and vanillylidenacetone and gallic acid were selected from the third cluster. The compounds were added to the cultivation medium at their respective toxicity limit concentrations of 18.0 mM syringic acid, 18.0 mM dihydroferulic acid, 9.0 mM homovanillic acid, 11.0 mM 4 hydroxybenzoic acid, 4.2 mM vanillylidenacetone and 9.4 mM gallic acid.

**Figure 3:**
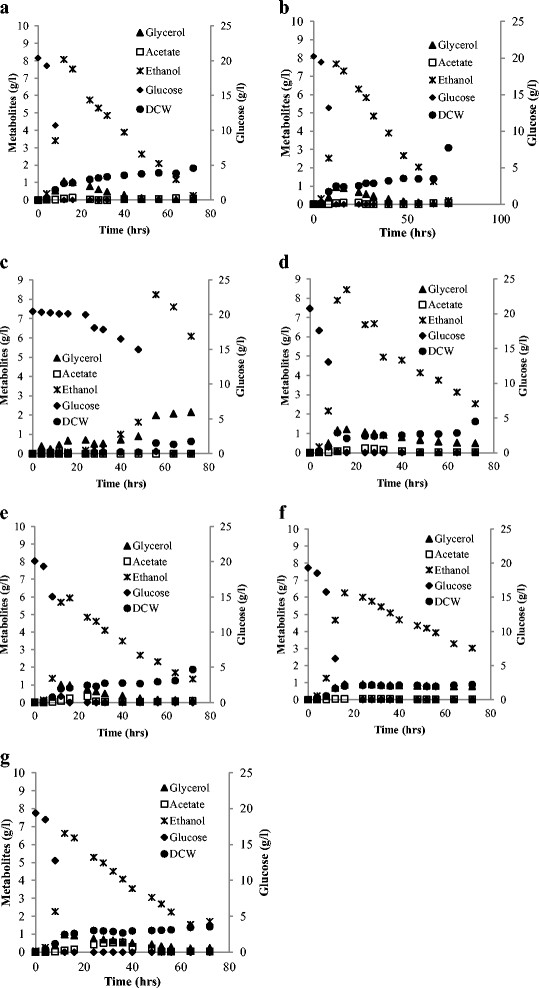
**Metabolite profiles representatives of** ***Saccharomyces cerevisiae*** **cultivations in the presence of: a. YMMM; b. syringic acid; c. dihydroferulic acid; d. homovanillic acid; e. 4-hydroxybenzoic acid; f. vanillylidenacetone and g. gallic acid.**

Glucose consumption was particularly delayed in dihydroferulic acid cultivations (Figure 3). No significant difference in glucose consumption was observed between any of the cultures containing syringic, homovanillic, 4-hydroxybenzoic, gallic acid or vanillylidenacetone, and the control (Figure 3). Ethanol assimilation after glucose depletion during the respiratory growth phase was slowed down for all cultures with the phenolic compounds except for cultures containing syringic acid.

**Figure 4:**
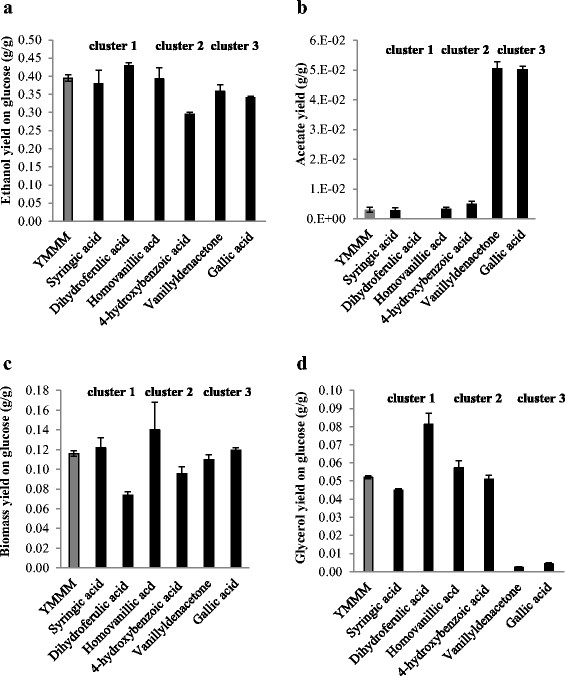
**Intra-cluster comparison of the effects of phenolic compounds on: a. ethanol yield; b. acetate yield; c. biomass yield; and d. glycerol yield from glucose.** Syringic and dihydroferulic acids were selected from cluster 1, homovanillic and 4-hydroxybenzoic acid from cluster 2, vanillylidenacetone and gallic acid from cluster 3.

Further comparison within each cluster was done based on the yields of ethanol, glycerol, acetate and biomass. Ethanol yield in dihydroferulic acid and syringic acid cultures were similar at 0.43 ± 0.01 (g/g) and 0.38 ± 0.03 (g/g) respectively (Figure 4). The yield of glycerol in dihydroferulic acid containing cultures was higher than in syringic acid containing cultures with yields of 0.081 ± 0.006 (g/g) and 0.045 ± 0.001 (g/g) respectively. The most outstanding difference between this pair however was that acetate was not found in dihydroferulic acid cultivations while acetate yield was 0.003 (g/g) in syringic acid cultures which was the same as that of the control (Figure 4).

Ethanol, acetate and biomass yields in homovanillic acid cultures were significantly different to 4-hydroxybenzoic acid cultures. Ethanol yield at 0.39 ± 0.03 (g/g) and biomass at 0.14 ± 0.03 (g/g) were higher in homovanillic acid containing cultures compared with 0.3 ± 0.01 (g/g) and 0.096 ± 0.007 (g/g) respectively for ethanol and biomass yields in 4-hydroxybenzoic acid. Acetate yield was lower in homovanillic acid cultures at 0.003 ± 0.0005 (g/g) compared to 0.005 ± 0.001 (g/g) in 4-hydroxybenzoic acid. However, glycerol yields of homovanillic and 4-hydroxybenzoic acids were similar at 0.057 ± 0.004 (g/g) and 0.051 ± 0.002 (g/g) respectively. Results for vanillylidenacetone and gallic acid (cluster 3) proved very consistent for ethanol, biomass, acetate and glycerol yields (Figure 4). A significant difference was observed between glycerol and acetate yields in the third cluster (vanillylidenacetone and gallic acid) and those in the other two clusters and the control. Glycerol yield in cluster 3 was 10 times lower at 0.002 ± 0.0002 (g/g) for vanillylidenacetone and 0.004 ± 0.0006 (g/g) for gallic acid cultures, and acetate yield was higher by 10 times at 0.051 ± 0.002 (g/g) for vanillylidenacetone and 0.05 ± 0.001 (g/g) for gallic acid than in both YMMM and the other two clusters (Figure 4). Overall ethanol yield in dihydroferulic acid was the highest at 0.43 (g/g) while 4-hydroxybenzoic acid had the lowest ethanol yield and the highest acetate yields of all cultures. The similarities in effect of each clustered pair of phenolic compounds on yeast metabolism indicate that compounds in the same cluster have similar inhibitory effects on yeast.

## Discussion

In this study, we classified 13 different phenolic compounds commonly found in lignocellulosic hydrolysates according to their effect on *S. cerevisiae* growth. In particular, we showed that (i) the concentration that induces inhibitory effects is highly variable among phenolic compounds and it does not follow the order of phenolic aldehydes and ketones of being the most toxic, followed by acids and alcohols, respectively (Almeida *et al*. [2007a], Klinke *et al*. [2003]) (ii) the influence of phenolic compounds on *S. cerevisiae* growth follows three major patterns; (iii) different compounds have distinct effect not only on biomass formation but also on the production of ethanol, acetate and glycerol.

**Figure 5:**
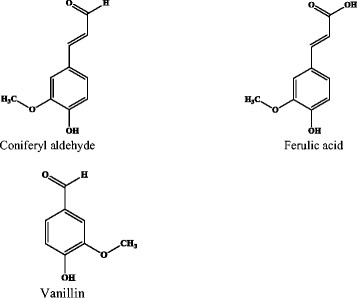
Structures of coniferyl aldehyde, ferulic acid and vanillin.

Phenolic compounds have often been grouped and ordered as aldehydes, phenolic ketones, phenolic acids and phenolic alcohols, and their potency as inhibitors has largely been believed to reflect the same order. Phenolic aldehydes have generally been regarded as the most inhibitory while phenolic acids and alcohols tend to be seen as the least toxic (Almeida *et al*. [2007a], Klinke *et al*. [2003]). In this study however, we demonstrated that the toxicity of phenolic compounds does not follow the assumed order in the subset of compounds we selected and is not dependent only on the recognised aldehyde, carboxylic acid, alcohol and ketone functional groups. Based on our results, we speculate that the inhibitory effects of phenolic compounds is a function of the combination of the occurrence of functional side groups (such as the methoxy and hydroxyl groups) and occurrence of unsaturated bonds in the structure of the compounds regardless of the categorization of the compounds as aldehydes, acid, alcohols or ketones. An example that supports this speculation is the different toxicities of coniferyl aldehyde (1.1 mM), ferulic acid (1.8 mM), and vanillin (9.7 mM) see Figure 5. Our results thus show that although coniferyl aldehyde is the most toxic at 1.1 mM, ferulic acid is more toxic at a toxicity limit of 1.8 mM than vanillin which is an aldehyde with a toxicity limit of 9.2 mM. The major difference between vanillin and coniferyl aldehyde is the occurrence of 2 extra carbon atoms sharing a double bond and linking the aldehyde group to the aromatic ring in coniferyl aldehyde. Ferulic acid also possesses 2 extra carbon atoms sharing a double bond and linking the carboxylic acid group to the aromatic ring. We speculate that these chemical features significantly contribute to the toxicity of coniferyl aldehyde and ferulic acid, in line with earlier findings that the occurrence and positions of functional side groups as well as the presence of unsaturated carbon to carbon bonds influence the biological reactions and inhibitory activities of phenolic compounds in bacteria as well as their antioxidant activity in human (Ramaswam *et al*. [1972], Rice-Evans *et al*. [1996]).

Three distinct growth patterns among the thirteen different phenolic compounds screened was observed, suggesting that compounds belonging to the same cluster display similarity in mechanisms of inhibition mechanisms. The similarity of ethanol yields between compounds representing cluster 1, acetate and glycerol yields in cluster 2 and the strong correlation of effects of vanillylidenacetone and Gallic acid (cluster 3) on ethanol, glycerol, acetate and biomass yields suggest that compounds belonging to the same cluster have similar inhibitory activity on yeast.

Phenolic compounds have been shown to reduce yields of ethanol and alter glycerol and acetate yields from *S. cerevisiae* fermentations. Studies by Ando *et al., * ([1986]), revealed that syringaldehyde, m-hydroxybenzoic acid and vanillic acid did not inhibit ethanolic fermentation while coniferyl aldehyde led to poor fermentation and drastically reduced ethanol yield. Larsson *et al*. ([2000]) also corroborated the severely inhibitory effect of coniferyl aldehyde in their study. Our results aligned with previous studies which strengthens our confidence in the toxicity ranking of all our tested phenolic compounds in which we found coniferyl aldehyde to be the most toxic phenolic compound. The influence of the phenolic compounds on yeast physiology was mostly visible through their impacts on glycerol, biomass and acetate yields among the compound clusters. Absence of quantifiable acetate production, poor growth and delayed glucose utilisation characterized the dihydroferulic acid cultivations. Known conditions that may prevent the accumulation of acetate in cultures include, disruption of acetaldehyde dehydrogenases, low formation of acetaldehyde coupled with effective oxidation of acetate by acetyl-coA-synthetase or the presence of low amount of glucose in cultures such that respirofermentative growth cannot take place (Postma *et al*. [1989]). The presence of ethanol (0.43 g/g) and glycerol did confirm a respirofermentative growth for the yeast under these cultivation conditions. Speculatively, the apparent absence of acetate in the cultivation may resemble a situation where low activity of Cytosolic Mg^2+^ and Mitochondrial K^+^ acetaldehyde dehydrogenases Ald6p and Ald4p is present. Mutants of *ALD6* have been shown to substantially reduce acetate production while significantly increasing glycerol production. Double mutants of Ald6p and Ald4p have been shown to have delayed growth, and delayed glucose consumption (Remize *et al*. [2000]), as observed in our dihydroferulic acid cultivation. It is therefore tempting to speculate that these two enzymes might be a direct or indirect target of ferulic acid, although this goes beyond the purpose of this article and deserves further investigation. Glycerol being a metabolite strongly associated with different types of stress in cells, the particularly high glycerol titre and yield in the cultivation of dihydroferulic acid is indicative of the cells being under significant stress from dihydroferulic acid although we have not defined the type of stress imposed at this stage of the study.

Although glucose consumption was delayed in dihydroferulic acid cultivations, ethanol yield was high and slightly superior to the ethanol yield in the control. Ethanol yields recorded in this study were high, ranging from 0.3 ± 0.01 (g/g) in 4-hydroxybenzoic acid cultures to 0.43 (g/g) in dihydroferulic acid cultures, we attribute this to the ability of the cells to adapt to the compounds and in certain cases convert some of them such as 4-hydroxybenzoic acid and dihydroferulic acid and eventually recover, thus bringing to attention and supporting findings of the natural ability of *Saccharomyces cerevisiae* to tolerate phenolic compounds (Stratford *et al*. [2007]).

In conclusion, different phenolic compounds often present in lignocellulosic hydrolysates have toxicity limits that are not necessarily similar even between phenolic compounds sharing the same functional groups. An example of this would be the significant difference between ferulic acid and *p*-coumaric acid which we discovered in this study to respectively possess toxicity limits of 1.8 mM and 9.7 mM. The experiments showed that phenolics rich substrates may be fermentable since fermentability depends on the concentration and the nature of phenolic compounds present in them. Indications also emerged from the present study that mechanisms of inhibition among phenolic compounds are dissimilar and may not be defined by the classes of phenolic compounds (aldehydes, acids, alcohols and ketones) as they are currently known. Further studies involving investigation of gene regulation and varying enzymatic studies are needed to draw conclusions on the specificity of phenolic compounds inhibition in *Saccharomyces cerevisiae*.

## Competing interests

The authors of this work declare that they have no competing interests.

## Authors’ contributions

Experimental design, work and writing of the manuscript were carried out by PTA. Experimental design and the subsequent manuscript were reviewed by MA. and LO. All authors read and approved the final manuscript.

## Additional files

## Supplementary Material

Additional file 1: Figure S1. Effects of increasing concentration of phenolic compounds on maximum specific growth rates with: a. vanillin; b. *p*-coumaric acid; c. vanillylidenacetone representing the effects of phenolic compounds in clusters 1, 2, and 3 respectively, created according to the observed growth profile. Click here for file

Additional file 2: Figure S2. Effects of increasing concentration of phenolic compounds on final optical densities of cultivations with: a. vanillin; b. *p*-coumaric acid; c. vanillylidenacetone representing the effects f phenolic compounds in clusters 1, 2, and 3 respectively, created according to the observed growth profile. Click here for file
