# Supplementary material for: The chemical nature of phenolic compounds determines their toxicity and induces distinct physiological responses in Saccharomyces cerevisiae in lignocellulose hydrolysates
Source: AMB Express. 2014 May 29;4:46. doi: 10.1186/s13568-014-0046-7 (PMC4052683; doi:10.1186/s13568-014-0046-7)
Supplement: Additional file 1: Figure S1. — Effects of increasing concentration of phenolic compounds on maximum specific growth rates with: a. vanillin; b. p-coumaric acid; c. vanillylidenacetone representing the effects of phenolic compounds in clusters 1, 2, and 3 respectively, created according to the observed growth profile. [file s13568-014-0046-7-S1.docx]

**a b**

**c**

**Figure S1.** Effects of increasing concentration of phenolic compounds on maximum specific growth rates with: **a.** vanillin; **b.** *p*-coumaric acid; **c.** vanillylidenacetone representing the effects of phenolic compounds in clusters 1, 2, and 3 respectively, created according to the observed growth profile.
